# Critical care beyond organ support: the importance of geriatric rehabilitation

**DOI:** 10.1186/s13613-024-01361-8

**Published:** 2024-08-22

**Authors:** Jeremy M. Jacobs, Michael Beil, Christian Jung, Sigal Sviri

**Affiliations:** 1 Department of Geriatric Rehabilitation and the Center for Palliative Care, Hadassah Medical Center and Faculty of Medicine, Hebrew University of Jerusalem, Jerusalem, Israel; 2 Department of Medical Intensive Care, Hadassah Medical Center and Faculty of Medicine, Hebrew University of Jerusalem, Jerusalem, Israel; 3 Department of Cardiology, Pulmonology and Angiology, University Hospital, Düsseldorf, Germany

Authors response [1, 2].

We thank the author for the important comment, which emphasizes the fact that many of the people aged 80 and beyond, admitted to ICU, will not be alive one year later [1]. The comment suggests that a “profession focused upon curing, and a society which is reluctant to discuss dying is a powerful complicity.” Dr Hillman continues to argue that the result of such complicity is the delivery of inappropriate intensive care to patients deemed to be terminally ill.

This observation is obviously of great importance, and the provision of intensive care to terminally ill patients is largely considered to be not only futile, but also counterintuitive. We would argue that the very same observation also underlines the pressing need for improved accuracy of triage prior to admission to ICU. Similarly, during ICU care, we would emphasize the need for heightened surveillance, identification and earliest possible intervention among very old patients for whom survival is likely and among whom there exists the potential for subsequent rehabilitation [2].

Dr. Hill chooses to emphasize the importance of appropriate triage and timely allocation of palliative and end of life care, particularly among patients presenting with a “geriatric syndrome”. We would point out that prognostication is notoriously difficult with advancing age [3], becoming increasingly complex as numerous geriatric syndromes and concepts come into play, and far more subtle than simply the binary recognition of the presence or absence of a geriatric syndrome. Thus, the importance of preexisting functional and comorbid status, alongside cognitive, physical, sensory, locomotor, psychological capacity are all elements to be considered in the care of older people, both within and outside of the ICU setting [4, 5]. In attempting to predict survival and recognize subsequent rehabilitation potential, it is the fine balance between the final common pathways of frailty and resilience that needs to be accurately assessed. This remains the primary focus of our review, which attempts to emphasize the complexity of this process and to provide simple tools to aid in accurate assessment. Furthermore, the importance of time is introduced, both as an element of the patient’s clinical “momentum” to assist decision making, as well as the importance of earliest possible multidisciplinary intervention where appropriate.

As pointed out, the rate of favorable outcomes among very old people following ICU have been found to be lower than in younger individuals. Thus, it is important to have an accurate prognostic assessment to identify those patients for whom timely geriatric intervention and personalized rehabilitation care will be beneficial.
